# Antiangiogenic effects of oridonin

**DOI:** 10.1186/s12906-017-1706-3

**Published:** 2017-04-04

**Authors:** Lili Tian, Kangjie Xie, Donglai Sheng, Xiaoqing Wan, Guofu Zhu

**Affiliations:** 1 Traditional Chinese medicine pharmacy, Zhejiang Hospital, No. 12 Lingyin Road, Xihu District, Zhejiang, Hangzhou China; 2 Anesthesia Department, Zhejiang Cancer Hospital, No. 38 Guangji Road, Gongshu District, Zhejiang, Hangzhou 310002 China; 3 Institute of Developmental and Regenerative Biology, Hangzhou Normal University, No. 16 Xuelin Street, Jianggan District, Zhejiang, Hangzhou 310036 China; 4 School of Pharmacy, Shanghai University of Traditional Chinese Medicine, No. 1200 Cailun Road, Pudong New District, Shanghai, 201203 China

**Keywords:** Oridonin, Angiogenesis, HUVECs, Zebrafish, Mouse

## Abstract

**Background:**

Oridonin, the major terpene found in *Rabdosia rubescens* (Henmsl.) Hara, is widely used as a dietary supplement and therapeutic drug. Oridonin has been proven to possess good anti-tumour activity, but little is known about its effect on angiogenesis. The aim of this study was to investigate the antiangiogenic effects of oridonin in vivo and in vitro and prove that oridonin anti-tumour activity is based on suppressing angiogenesis.

**Methods:**

In vitro, the antiangiogenesis effect was studied by proliferation, apoptosis, migration, invasion, and tube formation experiments on human umbilical vascular endothelial cells (HUVECs). In vivo, using the Tg (fli1: GFP) zebrafish model, the embryonic vasculogenesis and postnatal regeneration were evaluated. The vascular endothelial growth factor (VEGF) signalling pathway gene expressions were assessed by reverse transcription-polymerase chain reaction (RT-PCR). Furthermore, the inhibition effects on tumour growth and metastasis were observed using a xenograft zebrafish tumour model and xenograft nude mouse tumour model. Angiogenesis was assayed by immunostaining with cluster of differentiation 31. Importantly, the proteins were identified as being differentially expressed in an in vivo model by two-dimensional electrophoresis-mass spectrometry (2D–MS) and western blot (WB).

**Results:**

The results indicated that oridonin inhibited HUVEC proliferation, migration, invasion, and tube formation and induced cell apoptosis. Oridonin inhibited zebrafish angiogenesis during embryonic development and tail fin regeneration. RT-PCR showed that oridonin decreased the VEGFA, VEGFR2, and VEGFR3 expressions in zebrafish, while the TP53 expression increased. Moreover, oridonin had strong effects on tumour growth and metastasis in vivo. 2D–MS identified a total of 50 proteins differentially expressed (17 up-expressed, 28 down-expressed). Lastly, WB showed that Claudin 1, Claudin 4, and Claudin 7 were closely related to tumour growth and metastasis.

**Conclusion:**

This study demonstrated that oridonin could inhibit tumour growth and metastasis, which mainly based on oridonin antiangiogenic effects. Claudin 1, Claudin 4, and Claudin 7 were the main contributors to the mechanism.

## Background

Angiogenesis, new blood vessel formation, is a significant step in biological and pathological processes. The identification of small molecules that block angiogenesis and are safe has been a challenge in drug development. Specific anti-angiogenic agents have arisen as an attractive therapeutic approach for the treatment of angiogenesis-dependent diseases, especially tumour metastasis [1–4]. The Vascular endothelial growth factors (VEGFs), especially VEGF-A, are key angiogenic factors. The VEGF receptor VEGFR2 is the primary receptor mediating the angiogenic activity of VEGF through distinct signal transduction pathways [5, 6]. Anti-angiogenic drugs, e.g., bevacizumab (Avastin®), inhibit VEGF signalling by blocking either VEGF ligands or VEGFRs. However, therapy-associated problems and severe adverse events are desirable to be overcome [7, 8]. To exploit safer and efficient agents, researchers have investigated natural products. In addition to their direct effect upon cancer cells, a number of herbal remedies have been identified to suppress angiogenesis and thereby reduce tumour growth. Certain Chinese herbal medicines contain natural anti-angiogenic compounds that need to be proven effective and safe for human use [9–12].

Oridonin, an effective diterpenoid component, has been proven to possess anti-tumour activity [13–18]. Oridonin inhibits the formation of capillary-like networks, indicating that it exhibits anti-angiogenesis activity [19]. Dong et al. proved the oridonin inhibits tumour growth and metastasis through anti-angiogenesis by blocking the Notch signalling [20]. Liu et al. indicated that the oridonin anti-tumour effect was mainly based on its anti-proliferation and anti-angiogenesis [18]. However, the anti-tumour effect of oridonin based on its inhibition angiogenesis and its mechanism has still not been fully elucidated in vivo.

In the present study, we chose the Tg (fli1: GFP) zebrafish model, and investigated how oridonin inhibits angiogenesis. The anti-tumour effect of oridonin in vivo was observed in a xenograft zebrafish tumour model and xenograft nude mouse tumour model. Importantly, 2D–MS, RT-PCR, and WB identified anti-tumour mechanisms based on the inhibition of angiogenesis in vivo.

## Methods

### Reagents

Oridonin (No: 1126YA13, purity more than 98%) was purchased from Shanghai Yuanye Bio-Technology Co., Ltd., in China. A 100 g/L stock solution of oridonin was diluted in 100% dimethyl sulfoxide (DMSO) and stored at 4 °C in darkness. A serial dilution was made in 100% DMSO that was 1000 times more concentrated to allow for a 1:1000 dilution with EM to create a serial dilution with a final DMSO concentration of 0.1%. HepG2-Luciferase cells were purchased from Shanghai Biomodel Organism Science & Technology Development Co., Ltd. Cell Tracker CM-Dil (No: 1,583,101) was purchased from Shanghai Qianchen Biological Technology Co., Ltd. RPMI1640 culture solution (No: 11,875,093) was purchased from Invitrogen. Annexin V-FITC (No: 556,547) and Matrigel (No: 354,234) were purchased from BD Pharmingen. Calcein-AM (No: 56,496), MTT (No: M5655), penicillin, streptomycin, DMSO, and tricaine methanesulfonate (MS-222) were obtained from Sigma. Bevacizumab (Avastin®) and Trizol (No: 11,667,165,001) were purchased from Roche. Anti-CD31, claudin-1 (No: 81,796), claudin-4 (No: 37,643), and claudin-7 (No: 17,670) were purchased from Sigma. Recombinant RNase (No: 2313A), Reverse TranScriptase M-MLV (No: 2641A), and SYBR Premix Ex TaqTM (No: RR420) were purchased from Takara. The other chemicals used in this study were of analytical grade.

### HUVEC culture, viability and apoptosis assay

Human umbilical vascular endothelial cells (HUVECs) (cell bank, SIBS, CAS.) were cultured in endothelial cell growth medium (ECGM): RPMI1640 medium supplemented with 10% foetal bovine serum, 100 U penicillin, and 100 U streptomycin, at 37 °C under a humidified 95%: 5% (*v*/v) mixture of air and CO_2_. When grown to 6 × 10^4^ cells/well, the cells were treated with DMSO (0.1%) or various concentrations of oridonin (20, 39, 78, 156, 312, 625, 1250, and 2500 μg/ml) for 24, 48, 72, and 96 h. The cell viability was determined by an MTT assay at 570 nm. At least three independent experiments at least were performed in triplicate.

HUVECs (2.5 × 10^5^ cells/mL) were treated without or with oridonin at various concentrations (39, 156, 625, and 2500 μg/ml) for 24 h. Then, the cells were harvested by trypsinization, washed twice with PBS, and labelled with a kit according to the directions and examined.

### HUVEC migration and invasion assay

HUVECs were cultured in ECGM, and 4 × 10^5^ HUVECs were seeded on Millicell treated without or with oridonin at various concentrations (39, 78, 156, and 312 μg/ml) for 24 h. Then, the cells that did not pass through were removed, and a crystal violet staining assay and acetic acid destaining were performed. The crystal violet medium was gathered up, and the assay was examined at 570 nm.

The cells were placed on a Transwell Building support, and the next day 2% BAS was added, and the cells were dried at 37 °C for 2 h and washed with PBS. The HUVECs were cultured in ECGM, and 4 × 10^5^ HUVECs were seeded on Millicell treated without or with oridonin at various concentrations (39, 78, 156, and 312 μg/ml) for 24 h. Then, the cells that did not pass through were removed, and a crystal violet staining assay and acetic acid destaining were performed. The crystal violet medium was gathered up, and the assay was examined at 570 nm.

### HUVEC tube formation assay

HUVECs were cultured in ECGM, and when the cells grew to 4 × 10^5^ cell/well, they were treated without or with oridonin at various concentrations (25, 100, and 400 μg/ml) for 24 h. Then, Matrigel was added, and the cells were dried at 37 °C for 6 h. Finally, Calcein-AM was added and the cells were incubated for 37 min in the darkness according to the directions and examined.

### Fish husbandry and embryo collection

Zebrafish (fli1: GFP) were obtained from Institute of Biochemistry and Cell Biology, SIBS, CAS. All fish were at the juvenile stage and were cultured in our laboratory until sexual maturation for spawning at standard laboratory conditions of 28 ± 0.5 °C on a 14:10 light/dark photoperiod in a recirculation system according to standard zebrafish breeding protocols [21]. The water supplied to the system was filtered by reverse osmosis (pH 7.0–7.5), and Instant Ocean® salt was added to the water to raise the conductivity to 450–1000 μS/cm (system water). The fish were fed twice a day with live artemias (Jiahong Feed Co., Tianjin, China).

Zebrafish embryos were obtained from adults in tanks with a sex ratio of 1:2 for female to male, and spawning was induced in the morning when the light was turned on. Embryos were collected within 0.5 h of spawning and rinsed in an embryo medium (EM: 0.137 M NaCl, 5.4 mM KCl, 0.25 mM Na_2_HPO4, 0.44 mM KH_2_PO4, 1.3 mM CaCl_2_, 1.0 mM MgSO_4_ and 4.2 mM NaHCO_3_) [21], and then incubated in Petri dishes at 28 ± 0.5 °C until chemical treatment. The fertilized embryos with normal morphology were staged under an SMZ 1500 dissection microscope (Nikon, Japan) according to the standard methods [22].

### Assessment of zebrafish embryo vascular changes and microscopy

The zebrafish embryos were treated with EM or various concentrations of oridonin (50, 100, and 200 μg/ml), and incubated in 96-well plates (1 embryo per well with 200 μl solution) at 28 ± 0.5 °C from 6 h post-fertilization (hpf) to 72 hpf. At 72 hpf, the larvae were removed from the 96-well plates and anesthetized with 0.03% MS-222 for 3–5 s until their bodies stopped moving. Then, the larvae were transferred to a glass slide, and the morphological and viability changes of the vessels were observed with a fluorescence inverted microscope (Nikon, Japan). Images were taken with NIS-Elements F2.3 (Nikon, Japan), and the number of complete intersegment vessels (ISVs) and the diameter of the blood vessels were obtained from the images. The assay was repeated at least three times independently with 10 embryos per group.

### Assessment of adult zebrafish caudal fin regenerative angiogenesis and microscopy

The zebrafish were normally divided into 3 groups and maintained at 28 ± 0.5 °C on a 14 h light/10 h dark cycle in tanks. The fish were anesthetized with 0.03% MS-222 for 2–3 min until the gills stopped moving and then promptly transferred to glass slides to amputate the caudal fins at the mid-fin level [23]. The fish were placed back to a recovery tank immediately and recovered within 3 min. EM and oridonin (50 and 150 μg/ml) were added to different tank at 28 ± 0.5 °C for 7 days post amputation (dpa), and the length of fin regeneration and regenerative blood vessels were obtained from images. The assay was repeated three times independently with 6 fins per group.

### Assessment of zebrafish larvae caudal fin regeneration and microscopy

The zebrafish larvae (48 hpf) were normally divided into 4 groups and maintained at 28 ± 0.5 °C on a 14 h light/10 h dark cycle in 6-well plates (10 larvae per well with 4 ml solution). The larvae were anesthetized with 0.03% MS-222 for 3–5 s until their body stopped moving, and then transferred to glass slides to amputate the caudal fins completely [24]. The larvae were immediately placed back and recovered within 3 min. EM and oridonin (10, 40, and 80 μg/ml) were added to different wells at 28 ± 0.5 °C for 4 dpa, and the length of the fin regeneration was obtained from images. The assay was repeated three times independently with 10 fins per group.

### RNA isolation and RT-PCR

After the 4 dpa exposure period, larvae of the same treatment were pooled together for the analysis of the VEGFA, VEGFR2, VEGFR3, and tumour protein p53 (TP53) expressions. Twenty larvae per group were homogenized in 1.0 ml Trizol reagent with a homogenizer (Polytron, Kinematica, Littau, Switzerland), and the total RNA was extracted according to the manufacturer’s protocol. The OD260/OD280 ratio, as well as the banding patterns on a 1% agarose gel, was routinely checked for the purity and integrity of the RNA sample. Reverse transcription was carried out using an M-MLV reverse transcriptase kit according to the manufacturer’s procedure. For RT-PCR, we used 2.0 μl from the reverse transcription product, amplified directly on a 7300 Real Time PCR System using a 20.0 μl SYBR reaction mixture. The primers (Biotechnology, Shanghai, China) were designed based on the cDNA sequences from the NCBI reported database (Table 1).

**Table 1:** Sequences of primers used in the RT-PCR

Gene name	Sequence of primers
VEGFA	Forward CAGCTGTCAAGAGTGCCTACATAC Reverse CATCAGGGTACTCCTGCTGAATTTC
VEGFR2	Forward TCACATGGTTTGGTAGAGGGATCTC Reverse GTGCAGTTGATCCTCTGCAAATGAG
VEGFR3	Forward TCTGTCGGATTTGGATTGGGA Reverse TTGGTGTTGTCAAGGGTGGG
TP53	Forward CTTGCCCCGTTCAAATGGTG Reverse TAGATGGCAGTGGCTCGAAC
β-Actin	Forward CGAGCAGGAGATGGGAACC Reverse CAACGGAAACGCTCATTGC

### Tumor model in zebrafish and anti-tumour effect

The zebrafish embryos were collected within 0.5 h of spawning and rinsed in EM, and then incubated in Petri dishes at 28 ± 0.5 °C in EM for 24 h. The PTU (0.2 mM) was added, and the embryos were incubated for an additional 24 h, and the prepared HepG2-Luciferase cells were labelled with DiI dye. At 54 hpf, the embryos were anesthetized by 0.03% (wt/vol) MS-222, and after 1 min, they were placed on slides. The tumor cells were counted at a concentration of 30 cells per nl to prepare for implantation, and the cell suspension was placed on ice during the entire microinjection procedure. The microcapillary glass needle was loaded with 4 μl of cell suspension. The needle was connected to the micromanipulator, and then the needle tip was gently inserted into the perivitelline space of the zebrafish embryo. 5 nl of the cell suspension containing approximately 150 cells was injected into the perivitelline space of each embryo, and the injected zebrafish embryos were transfered into PTU water.

The zebrafish tumour model was normally divided into 3 groups (control group, Avastin group, oridonin group) and maintained at 28 ± 0.5 °C on a 14 h light/10 h dark cycle in 6-well plates (10 larvae per well with 4 ml solution) for 7 days. The areas of tumour metastasis, heartbeats in 10 s and the body weight were obtained according to the images and tests. The assay was repeated three times independently.

### Protein analysis and identification

The number in each experimental group was 46. The samples of the control group and oridonin group were weighed, the lysis solution was added at 1:6 (*W*/*V*), and the solutions were homogenized and separated by centrifugation (11,000 pm, 2 s, 4 °C). After 3 min’ standing (4 °C), the solutions were centrifuged (4000 g) to obtain a supernatant for concentration by the Bradford method, and 100 μg total protein was prepared for isoelectric focusing (30 V 6 h, 60 V 6 h, 500 V 1 h, 1000 V 1 h, and 8000 V 20 h). Then, SDS-PAGE was performed, and the protein was stained by silver nitrate. After wet gel scanning, data analysis was performed using Image Master TM 2D Platinum software to obtain the differentially expressed proteins.

On the benchtop, we chosen certain points (50) and transferred them into 96 wells, added ddH_2_O (5 min ultrasound), acetonitrile (50 μl, dehydration), 10 mM DTT/25 mM NH_4_HCO_3_ (50 μl, 56 °C, 1 h), and 25 mM NH_4_HCO_3_ (50 μl, keep in darkness 45 min), and dehydrated and air-dried the solutions. Pancreatic enzyme liquid storage (10 ng/μl, 4 °C 30 min) and 25 mM NH_4_HCO_3_ (15 μl, 37 °C, 24 h) were added. TFA was added at a concentration of 0.1%, and the enzymatic hydrolysis reaction was terminated, followed by blending, and centrifugation. A mass spectrum sample was prepared for detection (Autoflex II MALDI-TOF/TOF, Smart Beam, Protomics HP, Cation mode), and the result was queried (Peptide Mass Fingerprint, Flex analysis 3.0, database search by http://www.matrixscience.com).

### Xenograft nude mouse tumour model

Male nude mice, 6 weeks old, were inoculated subcutaneously in the right flank with 5 × 10^6^ HepG2-Luciferase cells suspended in 50 μl PBS. When the tumour volume reached ∼150 mm^3^, the mice were randomly assigned to an oridonin treatment group (*n* = 6) or the control (DMSO) group (*n* = 6). The oridonin groups (2, 4, and 8 mg/kg) were administered daily with an intraperitoneal injection. The tumour volume was determined using digital Vernier calliper measurements and the formula A × B^2^ × 0.52, where A is the longest diameter of the tumour and B is the shortest diameter of the tumour. The inhibitory rate is calculated by V_0_-V/ V_0_ *100%, where V_0_ is the control group tumour volume and V is the oridonin group tumour volume.

### HE staining and immunostaining with CD31

Based on the primary tumour size, the mice were segregated into groups for the appropriate treatments. Starting on day 7, oridonin was injected intraperitoneally every 2 days. The lungs were dissected and fixed in 10% formaldehyde when the mice were agonal, and the paraffin-embedded lungs were cut for HE staining. The slides were examined under light microscopy, and pictures were taken at 200× magnification.

The tumours were harvested and excised, fixed in 4% neutral paraformaldehyde, and then embedded in paraffin and sectioned for the immunohistochemical analysis. The endothelial cells were identified by immunostaining with CD31 antibody. To evaluate the protein expression, semiquantitative image analysis on the sections was conducted by Image-Pro Plus software.

### RT-PCR and WB

The RT-PCR procedure was the same as seen earlier in this article. The primers (Biotechnology, Shanghai, China) were designed according to the cDNA sequences from the NCBI reported database, and are listed in Table 2. The WB procedure was as the follows. The zebrafish were treated under different conditions and lysed with lysis buffer. The protein samples were separated by electrophoresis on SDS-PAGE gels and transferred to a PVDF membrane. The blots were incubated with primary antibodies at 4 °C overnight, and after incubation with secondary antibodies, an enhanced chemiluminescence reagent was used for the signal detection. The data were quantified and normalized using Image software.

**Table 2:** Sequences of primers used in the real-time quantitative PCR

Gene name	Sequence of primers
Claudin 1	Forward CGATATTTCTTCTTGCAGGTCTGG
	Reverse CAAATTCGTACCTGGCATTGACT
Claudin 2	Forward CCAGAGAAATCGCTCCAACTACT
	Reverse GGCTGTAGGAATTGAACTCACTCTT
Claudin 3	Forward CCAACACCATTATCCGGGACTTC
	Reverse TAGACGTAGTCCTTGCGGTCGTA
Claudin 4	Forward AACATTGTCACCTCGCAGAC
	Reverse CAGGACACCGGCACTATCAC
Claudin 5	Forward TGATTGGCTGCGGCACGATGAC
	Reverse GCCCGCACGCCAGGATCAGAC
Claudin 6	Forward CTGACGCTAATCCCCGTGTG
	Reverse CCATTCCCCTCCACGTCAGA
Claudin 7	Forward ATGAGCTGCAAAATGTACGACT
	Reverse GCCATACCAGGAGCAAGCTAC
Claudin 8	Forward GGCCTTCATTGAAAACAACATC
	Reverse TGAAAGCCAAGAAGGACATCAC
Claudin 9	Forward TTCGACCGGCTTAGAACTGCT
	Reverse GTGAGTCGTACACCTTGCACT
Claudin 12	Forward ACTGCCTGATGTACGACACTA
	Reverse AAAAGACTGGCTCAAACTTCT
Claudin 18	Forward CATCTTTGCCCTGAAATGCATC
	Reverse ATTACACATAGTCGTGCTTGGA
β-Actin	Forward CACCCAGCACAATGAAGATCAAG
	Reverse TGTCAAGAAAGGGTGTAACGCAACT

### Statistical analysis

All data were reported as the means ± standard error (SEM). The *t*-test for independent analysis was applied to evaluate the difference between the treatment and control groups, and a value of *P* < 0.05 was considered statistically significant.

## Results

### Oridonin inhibited HUVEC proliferation

**Fig. 1:**
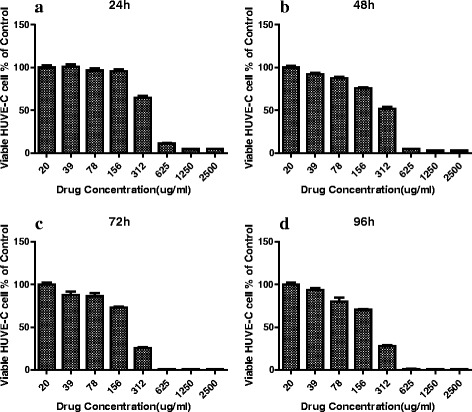
Effect of oridonin on HUVEC viability. HUVECs were treated with various concentrations (20–2500 μg/ml) of oridonin, and viability was determined by MTT assay at 24, 48, 72, and 96 h. **a** HUVEC viability at 24 h; **b** HUVEC viability at 48 h; **c** HUVEC viability at 72 h; **d** HUVEC viability at 96 h

According to preliminary experiments, we checked the HUVEC activity at 24, 48, 72, and 96 h. The MTT results showed that oridonin inhibited the HUVEC proliferation compared with those that were DMSO-treated (0.1%). The higher the concentration of oridonin was, the stronger the effect was (Fig. 1). The IC_50_ values were 272, 153, 121, and 109 μg/ml at 24, 48, 72, and 96 h respectively.

### Oridonin induced HUVEC apoptosis

**Fig. 2:**
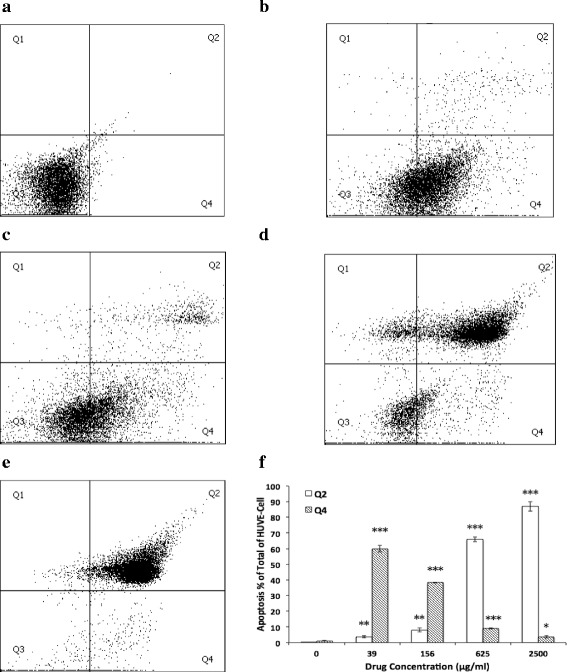
Effect of oridonin on HUVEC apoptosis. HUVECs were treated with various concentrations (39–2500 μg/ml) of oridonin, and apoptosis was determined by FAC assay for 24 h. **a** control group; **b** 39 μg/ml oridonin group; **c** 156 μg/ml oridonin group; **d** 625 μg/ml oridonin group; **e** 2500 μg/ml oridonin group; **f** HUVEC apoptosis for Q2 and Q4 comparison. The asterisks indicate statistically significant differences for oridonin groups compared with the control group (*P < 0.05, **P < 0.01, ***P < 0.001)

FACS detected the apoptosis of HUVECs, and the results showed that oridonin induced HUVEC apoptosis mainly in the stages of Q2 and Q4; the Q2 area for the late apoptosis of the cells and the Q4 area for the early apoptosis. Within the scope of concentration of the experimental groups, the higher the oridonin concentration, the stronger the effect was compared with the DMSO-treated group (0.1%) (Fig. 2).

### Oridonin inhibited HUVEC migration and invasion

**Fig. 3:**
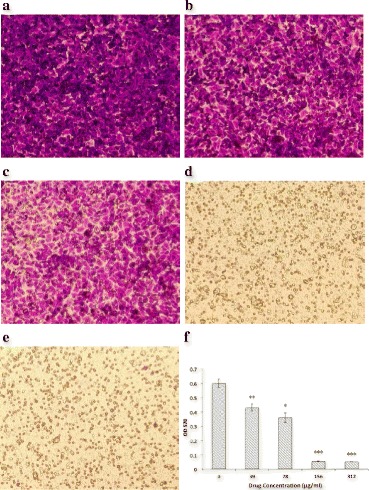
Effect of oridonin on HUVEC migration. HUVECs were treated with various concentrations (39–312 μg/ml) of oridonin. **a** control group; **b** 39 μg/ml oridonin group; **c** 78 μg/ml oridonin group; **d** 156 μg/ml oridonin group; **e** 312 μg/ml oridonin group; **f** OD570 comparison. The asterisks indicate statistically significant differences for oridonin groups compared with the control group (*P < 0.05, **P < 0.01, ***P < 0.001)

**Fig. 4:**
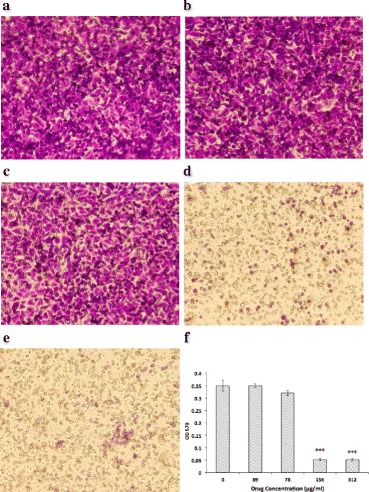
Effect of oridonin on HUVEC invasion. HUVECs were treated with various concentrations (39–312 μg/ml) of oridonin. **a** control group; **b** 39 μg/ml oridonin group; **c** 78 μg/ml oridonin group; **d** 156 μg/ml oridonin group; **e** 312 μg/ml oridonin group; **f** OD570 comparison. The asterisks indicate statistically significant differences for oridonin groups compared with the control group (***P < 0.001)

The transwell method detected human umbilical vein endothelial cell migration and invasion, and the results showed that oridonin inhibited HUVEC migration and invasion compared with DMSO- treated (0.1%) HUVECs. Within the scope of concentration of the experimental groups, with the higher oridonin concentrations, the inhibition effect was enhanced (Figs. 3 and 4).

### Oridonin inhibited HUVEC tube formation

**Fig. 5:**
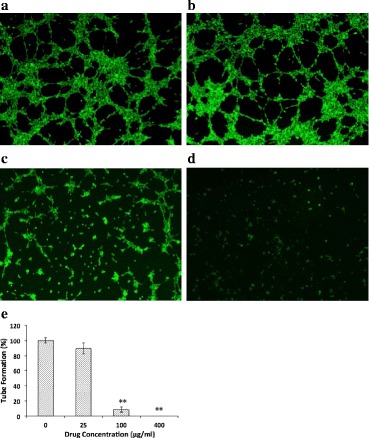
Effect of oridonin on HUVEC tube formation. HUVECs were treated with various concentrations (25–400 μg/ml) of oridonin. **a** control group; **b** 25 μg/ml oridonin group; **c** 100 μg/ml oridonin group; **d** 400 μg/ml oridonin group; **e** tube formation rate comparison. The asterisks indicate statistically significant differences for oridonin groups compared with the control group (**P < 0.01)

The results showed that oridonin inhibited the HUVEC tube formation compared with the DMSO-treated (0.1%) group. Within the scope of the low concentration to high concentration, the oridonin showed an increasing inhibition (Fig. 5).

### Oridonin inhibited angiogenesis during embryonic development

**Fig. 6:**
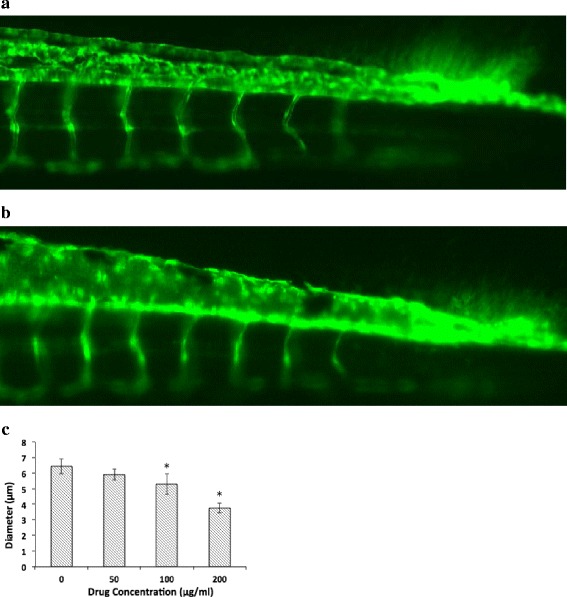
Antiangiogenic effects of oridonin in the zebrafish embryos. The average diameters of three blood vessels from the end of the tail were measured and calculated. **a** control group; **b** 200 μg/ml oridonin group; **c** Diameter length comparison. The asterisks indicate statistically significant differences from the control group (*P < 0.05)

Tg (fli1: GFP) zebrafish embryos were treated with EM or different concentrations of oridonin (50, 100, and 200 μg/ml) from 6 hpf to 72 hpf. When the zebrafish were analysed at 72 hpf, a time period in which all ISVs in the EM-treated control group from 6 hpf to 72 hpf were fully extended and formed dorsal longitudinal anastomotic vessels, the oridonin-treated groups showed significant reductions in the diameter of the complete ISVs compared with those of the control group. The greatest reduction was observed in the embryos treated with 100 and 200 μg/ml of oridonin (Fig. 6). Furthermore, the number of vessel segments that were extended from the yolk sac extension to the caudal fin remained unchanged. These results demonstrated that oridonin inhibited the angiogenesis of the ISVs in zebrafish embryos.

### Oridonin inhibited caudal fin regeneration of the larvae

**Fig. 7:**
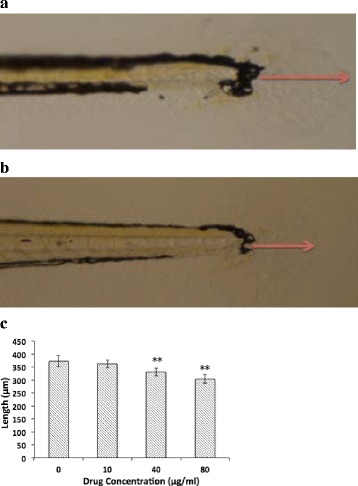
Antiangiogenic effects of oridonin on the larvae. The average lengths of the regenerated tissues were measured and calculated. **a** control group; **b** 80 μg/ml oridonin group; **c** Length comparison. The asterisks indicate statistically significant differences from the control group (*P < 0.05, **P < 0.01)

Figure depicts the lengths of regenerative caudal fins in the control group, which was treated with EM for 4 dpa and in the 80 mg L^−1^ oridonin group. Following oridonin treatment (10, 40, and 80 μg/ml) for 4 dpa, the lengths of the regenerative vascularized fin tissues dose-dependently decreased compared with those of the control group (Fig. 7).

### Oridonin inhibited caudal fin regeneration of adult zebrafish

**Fig. 8:**
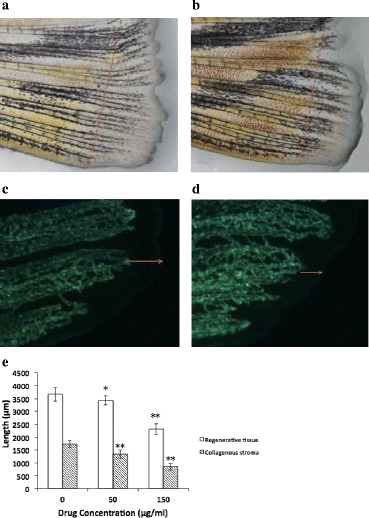
Antiangiogenic effect of oridonin in adult zebrafish. The average lengths of the regenerated tissues were measured and calculated. **a** control group (regular light); **b** 150 μg/ml oridonin group (regular light); **c** control group (fluorescent light); **d** 150 μg/ml oridonin group (fluorescent light); **e** Length comparison. The asterisks indicate statistically significant differences from the control group (*P < 0.05, **P < 0.01)

Figure depicts the lengths of the regenerative caudal fins in the control group, which was treated with EM for 7 dpa and in the 150 μg/ml oridonin group. Following oridonin treatment (50, and 150 μg/ml) for 7 dpa, the lengths of the regenerated vascularized fin tissues dose-dependently decreased compared with those of the control group (Fig. 8).

### Oridonin decreased VEGFA,VEGFR2,and VEGFR3 expression but increased TP53 expression

**Fig. 9:**
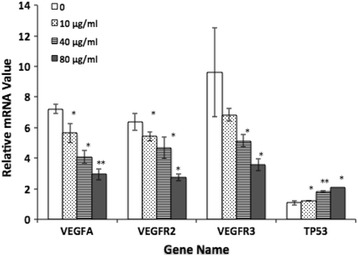
Gene expression comparison after exposure to various concentrations (10–80 μg/ml) of oridonin. The asterisks indicate statistically significant differences from the control group (*P < 0.05; **P < 0.01)

Zebrafish larvae were exposed to oridonin after 4 dpa, and the VEGFA and VEGFR2 levels were measured using RT-PCR. The results showed that the VEGFA, VEGFR2 and VEGFR3 gene levels dose-dependently decreased in the larvae after exposure to oridonin (10, 40, and 80 μg/ml) for 4 dpa, while the TP53 gene level dose-dependently increased (Fig. 9).

### Anti-tumour effect of oridonin in xenograft zebrafish model

**Fig. 10:**
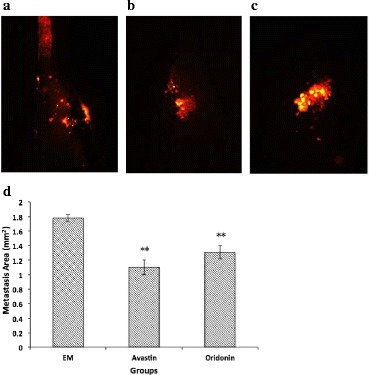
Tumour metastasis in xenograft zebrafish after 7 d exposure to EM, Avastin, and oridonin. **a** blank control group (EM); **b** positive control group (Avastin); **c** the group (oridonin); **d** Metastasis area comparison. The asterisks indicate statistically significant differences from the control group (**P < 0.01)

We found that oridonin impeded the tumour growth and metastasis in the xenograft zebrafish tumour model experiment. The anti-tumour effect of oridonin was weaker than that of Avastin (Fig. 10). However, oridonin had no effect on the heartbeats and body weights, while prolonged the survival times (Table 3). From the observation of the heartbeat and body weight on the 6th day, we proved some of the advantages of oridonin in applications.

**Table 3:** Comparison of the heartbeats, body weights, and survival times between the experimental groups

Group	Heartbeats	Body weights (mg)	Survival times (d)
Control	28.86 ± 0.69	1.49 ± 0.042	8.80 ± 0.92
Avastin	26.57 ± 0.79**	1.23 ± 0.055*	7.90 ± 0.32**
Oridonin	29.28 ± 0.76	1.47 ± 0.022	9.30 ± 0.83**

The asterisks indicate statistically significant differences from the control group (*P < 0.05, **P < 0.01)

### 2-D isoelectric focusing results

**Fig. 11:**
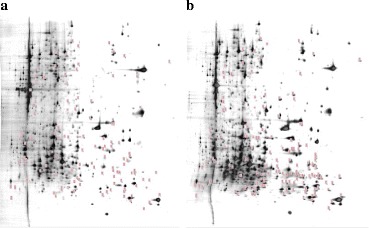
2-D isoelectric focusing results for the oridonin and control groups. **a** control group (EM); **b** drug group (oridonin)

We found that the oridonin groups (a, b, c) and the control groups (1,2,3) have very different protein quantities (Fig. 11).

### MS identification results

**Fig. 12:**
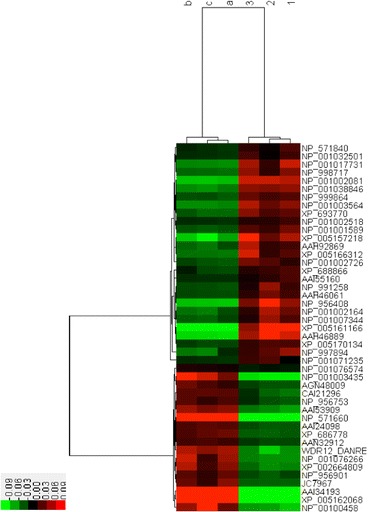
Cluster (heatmap) of MS identification results comparison between oridonin groups and control groups

We found that the oridonin groups (a, b, c) and the control groups (1,2,3) have very different protein quantities. A total of 50 proteins were found to be differentially expressed, of which 17 were up-expressed and 28 down-expressed (Table 4, Fig. 12).

**Table 4:** Protein comparison of MS identification results between oridonin groups (a, b, c) and control groups (1,2,3)

2-D ID	Score	Accession	AA	Protein Name	abc vs 123
51	78	NP_001076266	190	ubiquitin-conjugating enzyme E2 L3	Up
58	291	NP_571660	177	ferritin heavy chain	Up
88	135	AAI34193	185	Zgc:175,088 protein, partial	Up
114	132	CAI21296	253	ATPase, Na + \/K+ transporting, beta 1a polypeptide	Up
116	247	NP_956753	276	uncharacterized protein LOC393431/septin-2	Up
125	120	NP_001003435	296	39S ribosomal protein L15, mitochondrial precursor	Up
158	187	NP_001002081	127	small nuclear ribonucleoprotein Sm D3	Down
159	221	NP_999864	172	myosin regulatory light chain 12B	Down
163	142	NP_571840	209	claudin-4	Down
181	159	NP_001032501	213	high mobility group protein B2	Down
184	243	NP_001003564	210	DNA-directed RNA polymerases I, II, and III subunit RPABC1	Down
193	147	XP_688866	238	mitochondrial import inner membrane translocase subunit Tim21	Down
195	172	NP_001017731	256	uncharacterized protein LOC550426/C-factor	Down
202	279	NP_991258	256	eukaryotic translation initiation factor 4H isoform 2	Down
205	166	XP_005157218	279	transcriptional activator protein Pur-alpha	Down
215	200	NP_001002518	286	39S ribosomal protein L46, mitochondrial	Down
220	124	AAH46061	307	Ribonuclease H2, subunit A	Down
227	128	AAI55160	323	THO complex 6 homolog	Down
248	191	NP_001038846	349	phosphotriesterase-related protein	Down
263	166	NP_001002164	393	casein kinase II alpha 1 subunit	Down
267	274	NP_001076574	423	keratin 17	Down
269	188	AAH92869	433	Enolase 3, (beta, muscle)	Down
270	201	XP_005161166	412	tRNA pseudouridine synthase A, mitochondrial isoform X1	Down
276	165	NP_001001589	454	cytochrome b-c1 complex subunit 2, mitochondrial	Down
282	184	NP_956408	488	cleavage stimulation factor subunit 2	Down
287	176	AAH46889	449	Tubulin, alpha 4 like	Down
288	266	NP_001007344	469	sorting and assembly machinery component 50 homologue B	Down
305	178	NP_998717	483	6-phosphogluconate dehydrogenase, decarboxylating isoform 2	Down
314	226	NP_997894	492	UTP--glucose-1-phosphate uridylyltransferase	Down
376	115	XP_005166312	518	UDP-N-acetylhexosamine pyrophosphorylase isoform X1	Down
378	155	AAN32912	310	cathepsin, partial	Up
382	171	XP_005170134	521	non-syndromic hearing impairment protein 5	Down
391	196	AAI24098	327	Myef2 protein	Up
493	118	NP_956901	359	solute carrier family 25, member 1	Up
513	165	JC7967	441	Napor protein	Up
515	190	WDR12_DANRE	422	Ribosome biogenesis protein wdr12/WD repeat-containing protein 12	Up
534	144	XP_002664809	441	threonine--tRNA ligase, cytoplasmic-like, partial	Up
546	145	NP_001071235	612	3′-phosphoadenosine 5′-phosphosulfate synthase 2a	Down
599	145	XP_693770	964	ER membrane protein complex subunit 1 isoform X2	Down
658	177	NP_00100458	479	nuclear receptor coactivator 5	Up
802	175	AAI53909	481	Mybbp1a	Up
821	196	XP_005162068	513	glucose-6-phosphate 1-dehydrogenase isoform X4	Up
908	105	XP_686778	576	5′-nucleotidase domain-containing protein 3	Up
913	206	NP_001002726	1112	WD repeat and HMG-box DNA-binding protein 1	Down
1172	82	AGN48009	937	retinoic acid-inducible protein Ib	Up

### Anti-tumour effect of oridonin in xenograft tumour mouse model

**Fig. 13:**
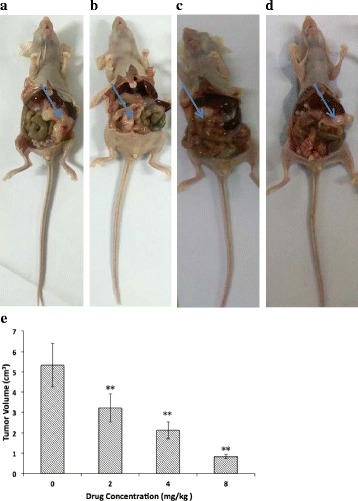
Anti-tumour growth effect in xenograft nude mouse. **a** control group; **b** 2 mg/kg oridonin group; **c** 4 mg/kg oridonin group; **d** 8 mg/kg oridonin group; **e** Tumour volumes comparison. The asterisks indicate statistically significant differences from the control group (**P < 0.01)

Oridonin had an anti-tumour effect in the xenograft tumour mouse model compared with the control group. Oridonin inhibited the tumour growth, and with the increasing concentration (2, 4, 8 mg/kg), the anti-tumour effect was stronger (Fig. 13, Table 5). In addition, in the process of the experiment, compared with the control group, the mice treated with oridonin exhibited an improved with mental state and dietary conditions.

**Table 5:** Comparison anti-tumor effects on experimental groups

Group (mg/kg)	Tumour weight (g)	Inhibitory rate (%)
0	1.24 ± 0.038	/
2	0.75 ± 0.12**	39.52
4	0.50 ± 0.088**	59.68
8	0.19 ± 0.054**	83.87

The asterisks indicate statistically significant differences from the control group (**P < 0.01)

### Haematoxylin & eosin staining and immunohistochemistry results

**Fig. 14:**
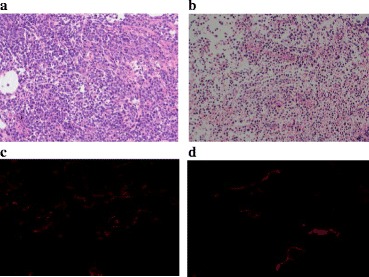
Tumour tissue section HE staining and CD31 fluorescent staining. **a** control group (HE); **b** oridonin group (HE); **c** control group (CD31); **d** oridonin group (CD31)

The HE staining of tissue sections of tumours from mice treated with oridonin showed that the cells were arranged with nest loading, interstitial, including visible more rules sample gland structure. The cells presented as circular, round or oval, with small nucleoli, nuclear fission, nuclear pulp ratio decrease, which prompted the cancer cells in the direction of high differentiation. The control group cell density was larger, with a diffuse distribution of a disordered arrangement that did not form nests or an adenoid structure. The cells were pleomorphic, round or oval, with a large nucleoplasm ratio, with deep nuclear staining deep, large and obvious nucleoli, and nuclear fission, which showed obvious necrosis. The mouse tumour tissue section CD31 fluorescent stained treated with oridonin showed that the blood vessel density in the tumour tissue was significantly less than that in the control group (*P* < 0.05), 36.62 ± 10.51 and 14.73 ± 8.20 respectively (Fig. 14).

### WB results

**Fig. 15:**
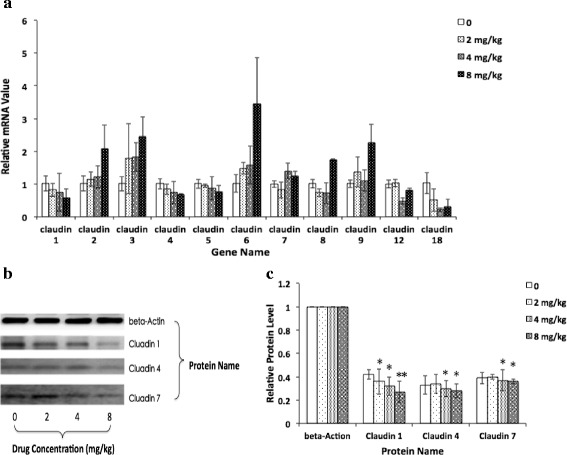
Claudin family genes and protein relative expressions upon exposure to various concentrations of oridonin for 7 d. **a** relative gene expression comparison; **b** protein levels in mouse tumour tissue sections detected by WB; **c** relative protein expression comparison. The asterisks indicate statistically significant differences from the control group (*P < 0.05, **P < 0.01)

The mouse tumour tissue sections were exposed to oridonin after 7 days, and the Claudin family gene expression levels were measured using RT-PCR. The results showed that the Claudin 1, Claudin 4, Claudin 5, Claudin 12, and Claudin 18 gene levels dose-dependently decreased (2, 4, and 8 mg/kg/d), and the Claudin 2, Claudin 3, Claudin 6, Claudin 7, Claudin 8, and Claudin 9 gene level dose-dependently increased. Further more, the WB results proved that the Claudin 1, Claudin 4, and Claudin 7 proteins were closely related to the mechanism of tumour growth and metastasis (Fig. 15).

## Discussion

At present, many cancer cases and deaths can be prevented through reducing the prevalence of risk factors, while increasing the effectiveness of clinical care, particularly for disadvantaged populations. We will facilitate the broader application of existing cancer control knowledge [25, 26]. Angiogenesis is required for tumour growth and metastasis. More than 90% of deaths from solid tumours are attributable to tumour metastasis [27]. Substantial research has been devoted to determining the effect of angiogenesis on tumour development and progression, and the mechanism of action of angiogenesis inhibitors is different from that of traditional chemotherapeutic agents and radiation therapy, providing them with potential in cancer management [28–31]. Moreover, researchers have focused on the health benefits of natural products that influence some steps in cancer-induced angiogenesis, especially in traditional Chinese medicine [32–34]. Identifying novel angiogenesis inhibitors can promote the discovery of new drugs for angiogenic diseases. The in vitro experiments in the present study showed that oridonin induced HUVEC apoptosis, and inhibited the proliferation, migration, invasion, and tube formation. The in vivo experiments demonstrated by vascular assays that oridonin inhibited angiogenesis in zebrafish embryos, suppressed the regenerated of the caudal fins of zebrafish larvae, and suppressed the regenerative angiogenesis of caudal fins in adult zebrafish. These results were consistent with those of a previous study, which found that oridonin could inhibit the formation of capillary-like networks in human dermal microvascular endothelial cells [19].

Phenotypic changes in angiogenesis are always involved in angiogenesis-related signalling pathways. VEGF signalling is mainly mediated by VEGFR2, and blocking the activity of VEGFR2 can limit angiogenesis [35, 36]. VEGFA is a key angiogenic factor, and VEGFR2, is the primary receptor mediating the angiogenic activity [5, 6]. VEGF signalling is a potential target of antiangiogenic therapy. VEGFR3 mainly participates in early blood vessels formation [37]. TP53 is one of the important tumour suppressor genes, which can make a gene stable and regulate cell growth, differentiation, and aging. TP53 is known as the “guardian gene” [38, 39]. Therefore, our study at least partially elucidates the mechanism of the antiangiogenic activity of oridonin, which may be related to the down-regulation of VEGFA, VEGFR2, and VEGFR3 expression, while up-regulation the TP53 expression.

We chose the xenograft zebrafish tumour model and observed the oridonin activity on tumour metastasis. The anti-tumour effect of oridonin was weaker than that of Bevacizumab. However, from the observations of the heartbeat, body weight and survival time, we demonstrated some advantages of oridonin in application. Moreover, 2D–MS identified a total of 50 proteins differentially expressed, of which 17 were up-expressed and 28 down-expressed. Moreover, among the 50 proteins, 12 proteins (3 up-expressed, 9 down-expressed) are closely related to tumour growth and metastasis: 5′-nucleotidase domain-containing protein 3, cathepsin, Napor protein (up-expressed); high mobility group protein B2, eukaryotic translation initiation factor 4H isoform 2/(EIF4H), transcriptional activator protein Pur-alpha, claudin-4, casein kinase II alpha 1 subunit, cytochrome b-c1 complex subunit 2, cleavage stimulation factor subunit 2, ER membrane protein complex subunit 1 isoform X2, and nuclear receptor coactivator 5 (down-expressed). In summary, these proteins can regulate the tumour cell proliferation, migration and growth by direct or indirect effects [40–49]. We chose the xenograft tumour mouse model and observed the oridonin activity on tumour growth and metastasis. The result verified the great anti-tumour effect of oridonin. Accordingly, the mice tumour tissue sections were subjected to HE staining and CD31 fluorescent staining, which showed the oridonin anti-tumour activity based on the inhibitory effect of angiogenesis.

**Fig. 16:**
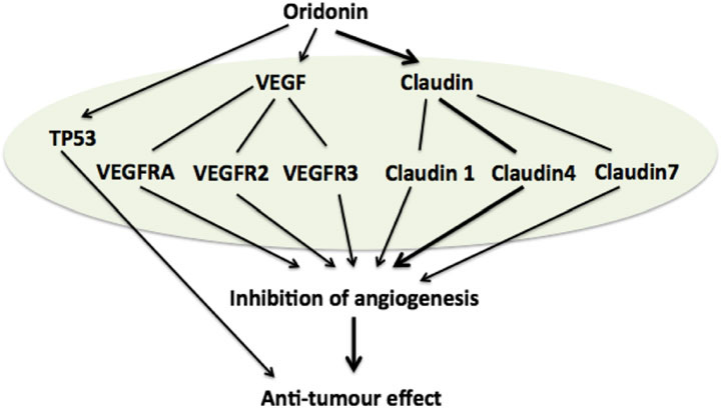
The different targets and signaling pathways regulated by oridonin. The pictorial summarized mechanism for inhibition of angiogenesis and anti-tumor effect by oridonin. Arrows indicate regulations by oridonin treatment in experimental results

Importantly, the Claudin-4 protein was focus of the research. Claudins are crucial structural and functional components of tight junctions, which are essential for holding endothelial and/or epithelial cells together. Claudin family proteins have a significant role in tumour growth, metastasis, and angiogenesis, and are highly expressed in many types of epithelial tumours [50, 51]. Claudin 4 is a transmembrane protein, which is often restricted to tight junction structures and has been shown to provide a barrier to paracellular diffusion. Research suggested that Claudin 4 plays an important role in tumour growth and malignancy via the control of cell proliferation, migration, apoptosis, and metastasis [52–54]. Significantly, Claudin 4 expression may be useful to distinguish switch/sucrose non-fermentable complex-deficient undifferentiated carcinomas from sarcomas [55]. Finally, our research showed that Claudin 1, Claudin 4, and Claudin 7 were closely related to tumour growth and metastasis. Claudin 1, Claudin 4 and Claudin 7 are down-expressed. Claudin 1 and Claudin 7 can be used in cancer diagnosis and treatment [56]. Claudin 4 has a correlation with tumour angiogenesis, and can be used as a treatment target protein, which was consistent with our research results [52, 53]. Moreover, the research conformed that Claudin 4 and VEGFA, VEGFR2, VEGFR3, and TP53 were all contributed to the anti-tumour effect based on the inhibition of angiogenesis (Fig.16). A meta-analysis indicates that Claudin-4 over-expression is associated with progress of gastric cancer and poor survival in gastric cancer patients [57]. Aberrant Claudin 4 expression plays an important role in the clinicopathological characteristics of gastric cancer [58]. Claudin 4 is thus a potent target for cancer therapy, and that an anti-Claudin 4 antibody is a promising candidate anticancer agent [59, 60].

## Conclusion

The present study has demonstrated that oridonin inhibited angiogenesis in vitro and in vivo. Further study indicated that the inhibited angiogenesis effect from oridonin might be based on the down-regulation of VEGFA, VEGFR2, and VEGFR3, and the up-regulation of TP53 gene expression. Claudin 1, Claudin 4, and Claudin 7 were the main proteins contributing to the oridonin anti-tumour effect based on the inhibition of angiogenesis.

## Abbreviations

2D–MS: Two-dimensional electrophoresis-mass spectrometer
CD31: Cluster of differentiation 31
DMSO: Dimethyl sulfoxide
dpa: Days post amputation
ECGM: Endothelial cell growth medium
EM: Embryo medium
HE: Hematoxylin and eosin.
hpf: Hour post-fertilization
HUVECs: Human umbilical vascular endothelial cells
ISVs: Intersegment vessels
RT-PCR: Reverse transcription-polymerase chain reaction
TP53: Tumor protein p53
VEGF: Vascular endothelial growth factor
WB: Western blot
